# Effects of Replacing Inorganic Sources of Copper, Manganese, and Zinc with Different Organic Forms on Mineral Status, Immune Biomarkers, and Lameness of Lactating Cows

**DOI:** 10.3390/ani15020271

**Published:** 2025-01-19

**Authors:** Manqian Cha, Xingjun Ma, Yunlong Liu, Shengyang Xu, Qiyu Diao, Yan Tu

**Affiliations:** 1 Sino-US Joint Lab on Nutrition and Metabolism of Ruminant, Key Laboratory of Feed Biotechnology of the Ministry of Agriculture and Rural Affairs, Institute of Feed Research, Chinese Academy of Agricultural Sciences, Beijing 100081, China; chamanqian@163.com (M.C.); liuyunlong9438@163.com (Y.L.); 82101211217@caas.cn (S.X.); diaoqiyu@caas.cn (Q.D.); 2 College of Animal Science and Technology, Gansu Agricultural University, Lanzhou 730070, China; 17899317590@163.com

**Keywords:** organic trace mineral products, methionine-hydroxyl-analog-chelated, amino acid complex, immune biomarker, lactating cows

## Abstract

(Objectives) The objectives of this study were to evaluate the effect of half-replacement of the supplementary sulfate sources of Cu, Mn, and Zn with methionine-hydroxy-analog-chelated (MHAC) mineral or amino-acid-complexed (AAC) mineral forms in diets on the mineral status, blood immune biomarkers, and lameness of lactating cows. (Methods) Sixty multiparous Holstein cows (158 ± 26 days in milk; body weight: 665 ± 52 kg; milk yield: 32 ± 7 kg/day) were randomly assigned into one of three dietary treatments (*n* = 20 per group): (1) MHAC: 50% replacement of sulfate minerals with MHAC forms. (2) AAC: 50% replacement of sulfate minerals with AAC forms. (3) S: 100% sulfate minerals (control). Their Cu, Mn, and Zn concentrations, blood immune biomarkers, and lameness were measured monthly. Repeated-measure mixed models were used to evaluate the effects on trace mineral status over time. As the responses with the MHAC and AAC forms were similar, the treatments were also analyzed as organic trace minerals (OTMs, combining the MHAC and AAC groups, *n* = 40) versus inorganic trace minerals (ITMs, the S group, *n* = 20). (Results) Cows supplemented with OTMs had higher concentrations of Cu and Mn in their serum (*p* ≤ 0.05), a higher hoof hardness (*p* ≤ 0.05), and a lower incidence of lameness compared to those with ITMs on d 90. There were no statistical differences (*p* > 0.10) in the concentrations of IgA, IgG, or ceruloplasmin, but there were significant differences (*p* = 0.03) in the concentrations of IgM in the serum as fixed effects of the diet treatments during the whole trial. On d 30 and 90, the serum IgA concentrations of the cows supplemented with OTMs tended to be higher (0.05 < *p* ≤ 0.10) than those in the cows supplemented with ITMs. (Conclusions) The half-replacement strategy showed that the MHAC and AAC sources of Cu, Mn, and Zn additives had similar effects on the production performance, blood immune biomarkers, and lameness of the lactating cows. The long-term replacement strategy with OTMs led to the enhancement of the trace mineral concentrations in their body fluids, blood immune biomarkers, and hoof health.

## Simple Summary

Copper, manganese, and zinc are crucial trace minerals for the productivity, immunity, and hoof health of dairy cows. Organic trace minerals, such as methionine-hydroxy-analog-chelated minerals and amino-acid-complexed minerals, exhibit higher bioavailability compared to that of inorganic trace minerals. However, the bioavailability of organic trace mineral additives differs substantially, resulting in inconsistent findings across studies. Yet the impact of replacing half of the sulfate copper, manganese, and zinc with methionine-hydroxyl-analog-chelated and amino-acid-complexed salts in lactating Holstein cows remains unexplored. This study assessed the effects of substituting half of the sulfate copper, manganese, and zinc with two organic trace mineral supplements on the lactation performance, mineral status, serum immune indicators, and lameness in cows.

## 1. Introduction

Trace minerals (TMs), though required in small amounts, are essential for the productivity, immunity, and hoof health of dairy cows [1,2]. Copper (Cu), manganese (Mn), and zinc (Zn) are among the most critical TMs, serving as cofactors for the enzymes involved in oxidative balance, protein synthesis, immune function, and other biological processes [3]. Proper dietary inclusion of TMs is crucial for maintaining structural, physiological, and catalytic functions in dairy cows. However, excessive mineral intake can cause environmental contamination and health issues, while deficiencies negatively affect performance [1,4]. Lameness is currently ranked as the third most important and economically demanding disease in the dairy industry.

The source of trace minerals greatly influences their absorption and utilization. Organic trace minerals (OTMs), such as methionine-hydroxy-analog-chelated (MHAC) minerals and amino-acid-complexed (AAC) minerals, offer higher bioavailability and are associated with improved immune response, hoof health, and production performance compared to inorganic trace minerals (ITMs) [5,6,7]. Lameness, a condition closely linked to mineral imbalance, is one of the most economically significant diseases in the dairy industry. Research has demonstrated the potential of OTMs to enhance udder health, reduce lameness, and improve reproductive performance [8,9,10]. Their efficient absorption and utilization allow for lower dietary inclusion rates, reducing environmental impact while maintaining optimal health and productivity [11,12]. Traditionally, ITMs were the primary sources of dietary TMs, but their inefficient utilization has shifted the focus toward OTMs [1,13,14]. Unlike ITMs, OTMs are protected from undesirable interactions in the gastrointestinal tract, increasing their bioavailability for absorption [4,12,15].

The Association of American Feed Control Officials (AAFCO) categorizes OTMs based on their ligands, such as amino acid complexes and chelates [13,16]. Covalent bonding within OTM structures protects the minerals from chemical reactions in the rumen, further enhancing absorption [13,14]. MHAC and AAC forms are the most common commercial OTMs that have been studied in dairy cows in terms of assessing their impacts on lactation performance, mineral metabolism, and immunity status, respectively. Byrne et al. (2022) reported a comprehensive review containing the most up-to-date information on the relative bioavailability of selected trace minerals (Cu, Fe, Mn, and Zn) used in ruminant nutrition [13]. Multiple studies have evaluated the effects of supplementing OTMs in MHAC or AAC forms on dairy or beef cow performance, and variable results were observed due to the diverse range of their experimental designs [6,17,18,19]. Moreover, partial replacement is a common practice in the dairy industry. Studies have shown that partially replacing ITMs (Co, Cu, Mn, and Zn) with OTMs during the transition period can improve mineral metabolism, immune function, and lactation performance [20,21]. Replacing 30–50% of dietary sulfate sources with MHAC or AAC forms has been linked to improved digestibility, plasma mineral concentrations, and milk yield [22,23,24]. It is worth noting that replacing 50% of dietary sulfate Cu and Mn with methionine hydroxy Cu and Mn increased the plasma Cu and Mn concentrations and tended to improve the apparent digestibility of NDF and the lactation performance of lactating cows [23,24]. However, the bioavailability of commercial OTM products varies significantly due to differences in pH-dependent stability and other factors, leading to inconsistent results across studies [16]. Despite growing evidence of the benefits of OTMs, there is still a limited understanding of how supplementation strategies affect cows’ performance, particularly in areas such as lameness and immune biomarkers.

This study aimed to evaluate the effects of replacing half of the supplementary sulfate sources of Cu, Mn, and Zn with MHAC or AAC forms in the diet on the production performance, blood immune biomarkers, and lameness of lactating cows. We hypothesized that the OTM replacement would enhance their trace mineral status, improve their immune response, and support hoof health. Our specific objectives were to further compare the effects of supplementary MHAC and AAC forms in the diet on the mineral status, blood immune biomarkers, and lameness of lactating cows.

## 2. Materials and Methods

This study was conducted from June to November 2022 at the Jinyuhaoxing Dairy Farm in Wuzhong City, Ningxia Hui Autonomous Region, China. The experimental protocol (IFR-CAAS20220423) was approved by the Institute of Feed Research of the Chinese Academy of Agricultural Science.

### 2.1. The Diets, Cows, and Experimental Design

Prior to the feeding trials, the basal levels of microminerals and TMs in the feed ingredients and the basal total mixed rations (TMRs) without any kind of supplemental TMs were measured (Figure S1). The average basal concentrations of Cu, Mn, and Zn in the basal TMRs (without any kind of supplemental TMs) were 5.18, 17.62, and 20.46 mg/kg, respectively. The sulfate form minerals of CuSO_4_·5H_2_O (25.0% Cu), MnSO_4_·H_2_O (31.8% Mn), and ZnSO_4_·H_2_O (35.5% Zn) were purchased from Changsha Rongqing Chemical Co., Ltd. (Changsha, China). The methionine-hydroxyl-analog-chelated forms of MHAC-Cu (12.0% Cu), MHAC-Mn (12.0% Mn), and MHAC-Zn (12.0% Zn) were purchased from XJ-BIO Manufacturing Facility (Liuyang, China). The amino acid complex forms of Availa-Cu (10.0% Cu), Availa-Mn (8.0% Mn), and Availa-Zn (12.0% Zn) were commercially available products from Zinpro Corporation (Shanghai, China). We declare that we have no financial and personal relationships with other people or organizations that could have inappropriately influenced our work.

**Figure 1:**
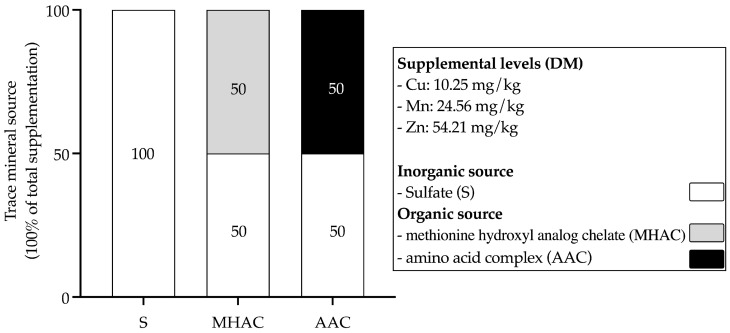
Schematic representation of experimental treatments. Treatments included 3 sources of supplemental Cu, Mn, and Zn. MHAC = replacing 50% of the sulfate form with 50% organic salts of trace minerals in methionine hydroxyl analog chelate form; AAC = replacing 50% of the sulfate form with 50% organic salts of trace minerals in amino acid complex form; S = 100% inorganic salts of trace minerals in sulfate form.

The experiment was designed as a randomized complete block design. Sixty multiparous healthy Holstein cows were first blocked according to their milk production. These blocks were then adjusted based on body weight (BW) and days in milk (DIM). The descriptive statistics on the experimental cows are shown in Table S1. At the beginning of the experiment, the cows in each group averaged (mean ± SD) a DIM of 158 ± 26 d, a BW of 665 ± 52 kg, and a milk production of 32 ± 7 kg/d and were randomly assigned (*n* = 20) to receive 1 of 3 dietary treatments. As depicted in Figure 1, the 3 treatments consisted of different supplements of Cu, Zn, and Mn in the experimental diets: (1) replacing 50% of the sulfate form with the methionine-hydroxyl-analog-chelated (MHAC) form; (2) replacing 50% of the sulfate form with the amino acid complex (AAC) form; and (3) using 100% ITMs in sulfate form (S). The farm and study personnel were blinded to the treatment assignments. The final TMRs (Table 1) were formulated using NDS Professional (developed by RUM&N., Milan, Italy), and this program was built around the latest version of CNCPS, v6.55. The inputs used for diet formulation included 23.8 kg/d of dry matter intake (DMI), 158 d DIM, a 32 kg/d milk production with 3.40% fat and 3.20% true protein, and a 665 kg BW.

The targeted amounts of Cu, Mn, and Zn in the final TMRs were 15.43, 42.18, and 74.67 mg/kg (on a DM basis), respectively. Therefore, the trace mineral and vitamin premixes (Table S2) were formulated to provide 10.25, 24.56, and 54.21 mg/kg (on a DM basis) of Cu, Mn, and Zn, respectively, for the TMRs to meet or exceed the requirements of NASEM (2021) [1]. The trace mineral and vitamin premixes were manufactured, packed, and transported to the trial farm by a commercial feed mill (Beijing Yonghe Rongda Nutrition Technology Co., Ltd., Beijing, China) every 10 days. The three treatment groups received the same diet except for the trace mineral and vitamin premixes. The analyzed mineral contents of the final TMRs are shown in Table 2. Cows in different groups were housed in separate free-stall barns equipped with waterers and fans and had free access to the experimental diets and water throughout this study. The cows were not under severe heat stress during the trial. The cows were hoof-bathed twice a week with 5% copper sulfate solution and once a week with 0.1% tetracycline solution to prevent lameness. The experimental diets were mixed using a 5 m^3^ mixer wagon (Storti Horizontal mixer wagon-Husky; Storti Co., Ltd., Beijing, China) and offered three times a day at 07:30 h, 14:30 h, and 19:30 h at 110% of the actual feed intake (on as as-fed basis) of the previous day, and refused feed was removed approximately 1 h before the morning feeding every morning. The feeding trial lasted for 90 days, and there were no lick bricks or other trace elements supplemented during the trial. Experienced veterinarians from the farm monitored the herd’s health throughout this study.

### 2.2. Data Collection, Sample Procedures, and Analytical Methods

#### 2.2.1. DMI and Diet Composition

The diets offered and refusals were weighed daily, and the average daily DMI of the dairy cows was determined by subtracting the refusals from the total as-fed TMRs (on a DM basis) during the experimental period. The average group DMI each week was calculated and is shown as the mean ± SD. Diets, drinking water, and feed refusals were collected consecutively for three days to yield one sample per week. The feed samples were analyzed for their DM (method 950.15), nitrogen (method 990.03), starch (method 996.11), ether extract (method 2003.05), and ash (method 924.05) according to the methods described by the Association of Official Analytical Chemists (AOAC) [25]. The contents of neutral detergent fiber (NDF) and acid detergent fiber (ADF) were analyzed using a fiber analyzer (A2000i; Ankom Technology, Fairport, NY, USA) [26].

#### 2.2.2. Milk Yield and Composition

Cows were milked 3 times daily (07:30, 14:30, and 19:30 h) using a 2 × 28-position parallel milking system (DeLaval (Tianjin) Co., Ltd., Tianjin, China), and each milk yield was recorded electronically for all cows throughout the trial. The milk composition was determined monthly (0, 30, 60, and 90 d). Individual milk samples from each cow were collected using the DeLaval milk sampling devices and mixed completely at a ratio of 4:3:3. One 50 mL aliquot of each composited milk sample was mixed with 1 potassium dichromate pill (0.5 g/pill) and stored at 4 °C until it was sent to the Ningxia Jinyuhaoxing Dairy Research DHI Laboratory (Wuzhong, Ningxia, China) for determination of the milk’s composition. The milk fat, protein, lactose, milk solids, and somatic cell count (SCC) were analyzed using an automated milk analyzer (Fossomatic^TM^ 7 DC; Foss Electric, Beijing, China). The 4% fat-corrected milk (4% FCM) yield was calculated as the milk yield (kg/d) × [0.377 + 0.116 × fat (%) + 0.06 × protein (%)] [27]. Another 150 mL aliquot of milk was stored at −80 °C for a later analysis of its mineral profile.

#### 2.2.3. Blood Sampling

Approximately 10 mL blood samples were collected from the coccygeal blood vessels into polyethylene terephthalate tubes specific to TM analysis at 07:00 h monthly (0, 30, 60, and 90 d). The blood samples were centrifuged at 3000× *g* (20 min, 4 °C) immediately after their collection and stored at −80 °C until the analysis. One set of serum was analyzed for the concentrations of blood immune indicators. The concentrations of IgA, IgG, IgM, ceruloplasmin, IL-4, IL-6, TNF-α, and T-AOC were determined using a multifunctional microplate reader (Synergy H1, Agilent BioTek, Santa Clara, CA, USA) with commercial ELISA kits (Nanjing Jiancheng Institute of Biological Engineering Co., Nanjing, China) with product numbers H108-1-2, H106-1-2, H109-1-2, A029-1-1, H005-1-2, H007-1-1, H052-1-2, and A015-2-1, respectively. Another set of serum was stored at −80 °C until a laboratory analysis of its mineral profile.

#### 2.2.4. Fecal and Urine Collection

Fecal and urine samples were collected on 0 ± 3, 30 ± 3, 60 ± 3, and 90 ± 3 d. During each sampling period, the samples were collected in a staggered schedule (every 6 h), with a total of four samples per sampling per cow. An approximately 250 g fecal sample was collected directly from the rectum each time from each cow. Meanwhile, approximately 100 mL urine samples were collected through stimulation of the cow’s vulva during each sampling from each cow. At the end of each sampling period, spot fecal or urine samples were pooled based on each cow to provide a composite sample. One 200 g aliquot of each pooled fecal sample was dried at 55 °C until no further weight loss in a forced-air oven and ground to pass through a 0.2 mm screen and then was analyzed for its acid-insoluble ash (AIA) content using the method from the International Organization for Standardization (ISO 9858:2002) [28]. One 100 mL aliquot of each composited urine sample was analyzed for its urine creatinine (CR) concentration using a commercial kit (Sigma no. 555; Sigma-Aldrich, Saint Louis, MO, USA). Another set of the pooled fecal and urine samples was aliquoted and stored at −20 °C until further analysis.

### 2.3. Evaluation of Hoof Health

Evaluations of hoof health were carried out on 0 ± 3, 30 ± 3, 60 ± 3, and 90 ± 3 d. The cow was fixed onto an electric hoof-repairing machine; then, hoof hardness was measured by pressing a durometer (Model DD-4 Digital Durometer Type D; Rex Gauge Limited, Lake Zurich, IL, USA) against the hoof wall at approximately 2 cm below the coronary band [29,30]. Meanwhile, samples of hoof keratin (0.5 cm × 2.5 cm × 0.5 cm) were collected from a bulbar zone which was 2 cm distal to the coronary band and 2 cm palmar/plantar to the dorsal wall of the hoof. These samples were then stored in small, sealed plastic containers [30,31]. Their gait scores were also assessed monthly according to a five-point locomotion scoring system as follows: 1 = a flat back, a smooth head bobbing frequency, easy joint activities, and a uniform stride; 2 = a slightly arched back, a smooth head bobbing frequency, mild joint stiffness, a slightly uneven gait, and no visible lameness; 3 = a light limp, an arched back, uneven head bobbing, joint stiffness, an uneven gait, and slight lameness; 4 = an obvious arched back, obvious head bobbing, joint stiffness, a hesitant gait, and obvious lameness; 5 = a severely arched back, very obvious head bobbing, joint stiffness, difficulty walking, and severe lameness [32,33]. Cows were considered lame when their score was ≥3 [31,32]. The evaluation and sampling were conducted by the research team and a professional hoof trimmer from the farm.

### 2.4. Determination of Mineral Concentration

All of the samples were analyzed for their concentrations using inductively coupled plasma mass spectrometry (Agilent 7500 Series ICP-MS; Agilent Technologies Inc., Wilmington, DE, USA) at the Institute of Animal Science Central Laboratory of the Chinese Academy of Agricultural Sciences (Beijing, China). The drinking water, urine, and serum samples were diluted at 0.5 mL in 10 mL with diluent working solution containing 1% HNO_3_, 1% isopropanol, 0.01% TritonX-100, 0.01% EDTA, and internal standards (25 μg/L Sc, 10 μg/L Ge, 5 μg/L Rh, and 2.5 μg/L Ir). The mineral concentrations in the samples were quantified using a multi-element standard curve using ICP-MS (Agilent 7500 Series ICP-MS, Agilent Technologies Inc., Santa Clara, CA, USA). Samples of the milk, hoof keratin, and feces were prepared and analyzed according to the methods by Mion et al. [6] and Daniel et al. [34]. Briefly, the samples were freeze-dried (Harvest Right Freeze Dryer, North Salt Lake, UT, USA) and microwave/acid-digested before their analysis. Approximately 0.5 g of dried sample was weighed in a Teflon vessel system (MARexpress, CEM, Matthews, NC, USA). A total of 4 mL of concentrated nitric acid (68%), 50 μL of Au solution (1000 ppm), and 0.25 mL of hydrochloric acid was added into the system and digested overnight. The digested samples were resuspended in 2 mL of deionized water. Appropriate standard reference materials and blanks were included with each batch run. The clear extract supernatants were further diluted and analyzed for their mineral concentrations using ICP-MS. The external calibration standards for the trace minerals were in the range of 0–100 μg/L. The concentrations of minerals in the milk and fecal samples were calculated based on the final volume after sample preparation and divided by their dry weight.

### 2.5. Statistical Procedures

Before the experiment, a power analysis was conducted to estimate the sample size based on previously published values [6,35,36]. According to the results of the power analysis, 60 cows in a completely randomized design (*n* = 20) were considered statistically sufficient with α (significance level) = 0.05 and the power = 0.80 to show biologically relevant treatment differences for milking performance and immune response. All of the data were analyzed using SAS (version 9.4; SAS Institute Inc., Cary, NC, USA). All of the data were tested for a normal distribution using the PROC UNIVARIATE procedure before the analysis. Outlier data were checked and excluded from the analysis using the PROC PLOT and PROC MEANS procedures based the Q-Q plots and studentized residuals. The best variance and covariance structure models were selected based on the values of the Akaike information criterion (AIC) and the Bayesian information criterion (BIC). The variables for DMI, milk yield, milk composition, TM concentrations, and blood indicators were analyzed as repeated measures using the PROC MIXED model as follows:

$$Y_{\mathrm{ijk}}{=\mu +\mathrm{Treat}}_{i}{+\mathrm{Cow}}_{j(i)}{+\mathrm{Time}}_{k}{+\mathrm{Treat}}_{i}\times {\mathrm{Time}}_{k}{+E}_{\mathrm{ijk}}$$

where *Y_ijkl_* = the dependent variable; *μ* = the overall mean; Treat*_i_* = the fixed effect of the *i*th diet treatments (*i* = 1 to 3); Cow*_j(i)_* = the random effect of the *j*th cow fed the *i*th diet (*l* = 1 to 20); Time*_k_* = the fixed effect of the *k*th sampling (milk yield, *k* = 1 to 13; milk composition, TM concentrations, and blood indicators, *k* = 1 to 4); Treat*_i_* × Time*_k_* = the interaction between the *i*th diet treatment and the *k*th sampling time; and *E_ijk_* = the residual error, assumed to be normally, identically, and independently distributed (NIID). In addition, differences among the three diet treatments were also analyzed using Tukey’s multiple comparisons.

The variables within one day or one sampling were analyzed using the PROC MIXED model, including the fixed effect of the diet treatments and the random effect of the cows, as follows:

$$Y_{\mathrm{ij}}{=\mu +\mathrm{Treat}}_{i}{+\mathrm{Cow}}_{j(i)}{+E}_{\mathrm{ij}}$$

The MHAC and AAC groups were combined into an organic trace mineral treatment (OTMs, *n* = 40), while S was analyzed as an inorganic trace mineral treatment (ITMs, *n* = 20). The contrast differences between OTMs and ITMs within one day or one sampling were analyzed using the above PROC MIXED model. The least-square means and standard errors of the means were reported throughout. Differences were considered significant at *p* ≤ 0.05 and tendencies considered at 0.05 < *p* ≤ 0.10.

## 3. Results

### 3.1. DMI, Milk Yield, and Milk Composition

**Figure 2:**
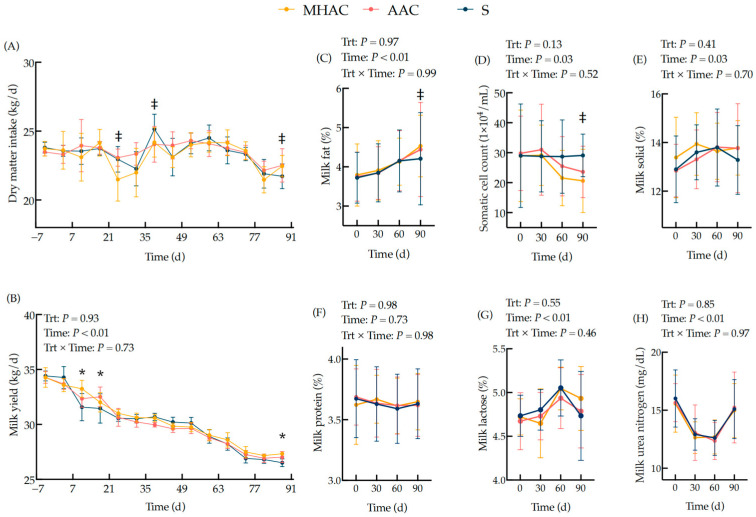
Effects of trace mineral sources on DMI (**A**), milk yield (**B**), milk fat content (**C**), somatic cell count (**D**), milk solids (**E**), milk protein (**F**), milk lactose (**G**), and milk nitrogen (**H**) of lactating cows. The DMI was shown as the mean of the group DMI ± SD weekly. Probability values for independent variables of interest: Trt = fixed effect of diet treatments; Time = fixed effect of sampling time; Trt × Time = interaction effect of diet treatments and sampling time. Within one day or one sampling, significant differences (*p* ≤ 0.05) were represented as follows: * ITM vs. OTM groups (contrast differences between inorganic trace mineral treatment (S, *n* = 20) and organic trace mineral treatments (MHAC and AAC, *n* = 40)); meanwhile, tendencies (0.05 < *p* ≤ 0.10) were represented by ‡.

There were no statistical differences (*p* > 0.10) in the milk yield, milk lactose, milk fat, milk protein, milk solids, milk urea nitrogen, milk protein yield, or milk fat yield of the three groups during the whole trial (Figure 2 and Table S3). Notably, a significantly higher average milk yield was observed for OTMs compared to ITMs on d 10, 15, and 90 (Figure 2B). Furthermore, the OTM group tended to have a higher milk fat content and a lower somatic cell count compared to that of the ITM group (0.05 < *p* ≤ 0.10) on d 90.

### 3.2. Cu, Mn, and Zn Concentrations

**Figure 3:**
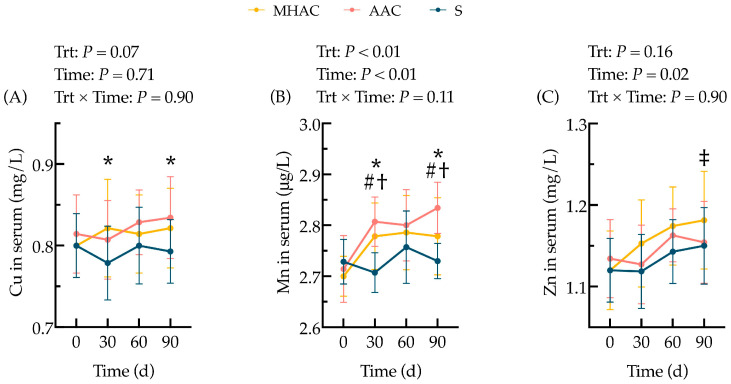
Effects of trace mineral sources on the concentration of Cu (**A**), Mn (**B**), and Zn (**C**) in the serum of lactating cows. Probability values for independent variables of interest: Trt = fixed effect of diet treatments; Time = fixed effect of sampling time; Trt × Time = interaction effect of diet treatments and sampling time. Within one day or one sampling, significant differences (*p* ≤ 0.05) were represented as follows: * ITM vs. OTM groups (contrast differences between inorganic trace mineral treatment (S, *n* = 20) and organic trace mineral treatments (MHAC and AAC, *n* = 40)); meanwhile, tendencies (0.05 < *p* ≤ 0.10) were represented by ‡. # = MHAC vs. S; † = AAC vs. S.

The concentrations of Cu, Mn, and Zn in the serum, milk, hoof keratin, urine, and feces of the lactating cows are shown in Table 3. For the concentration of trace minerals in the serum, there was a tendency for Cu (*p* = 0.07) and a significant difference for Mn (*p* < 0.01), but no differences in Zn (*p* > 0.10), in terms of fixed effects of the diet being observed during the whole trial. The cows supplemented with OTMs had higher concentrations of Cu in their serum (*p* ≤ 0.05) compared to those fed ITMs on d 30 and d 90 (Figure 3A). The cows supplemented with MHAC and AAC forms had higher concentrations of Mn in their serum than the supplemented cows from the S group (*p* ≤ 0.05) on d 30 and d 90 (Figure 3B). There was a tendency (0.05 < *p* ≤ 0.10) for the cows supplemented with OTMs to have a greater concentration of Zn in their serum on d 60 compared to those from the ITM group (Figure 3C).

Table 3 shows that half-replacement of the supplementary ITM forms of Cu, Mn, and Zn with the OTM forms in the diet significantly increased (*p* = 0.04) the Cu concentration in the milk and tended to increase the average Cu concentration in their serum and urine (0.05 < *p* ≤ 0.10). The concentrations of Zn in their serum, milk, urine, and feces did not differ among the treatments across the whole trial (*p* > 0.10).

### 3.3. Blood Immune Biomarkers

**Figure 4:**
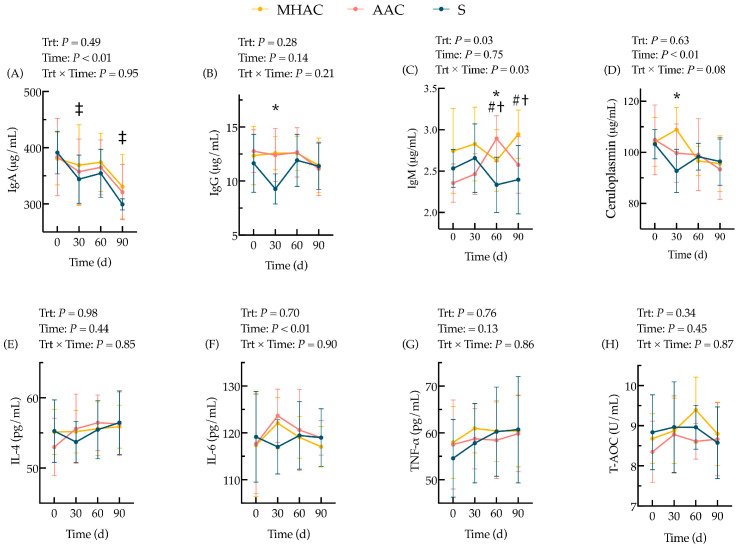
Effect of replacing inorganic trace minerals with organic trace minerals on blood IgA (**A**), IgG (**B**), IgM (**C**), ceruloplasmin (**D**), IL-4 (**E**), IL-6 (**F**), TNF-α (**G**), and T-AOC (**H**) of lactating cows. Probability values for independent variables of interest: Trt = fixed effect of diet treatments; Time = fixed effect of sampling time; Trt × Time = interaction effect of diet treatments and sampling time. Within one day or one sampling, significant differences (*p* ≤ 0.05) were represented as follows: * ITM vs. OTM groups (contrast differences between inorganic trace mineral treatment (S, *n* = 20) and organic trace mineral treatments (MHAC and AAC, *n* = 40)); meanwhile, tendencies (0.05 < *p* ≤ 0.10) were represented by ‡. # = MHAC vs. S; † = AAC vs. S.

The concentrations of the blood immune biomarkers in the cows are shown in Figure 4. There were no statistical differences (*p* > 0.10) in the concentrations of IgA, IgG, ceruloplasmin, IL-4, IL-6, TNF-α, or T-AOC but there were significant differences (*p* = 0.03) in the concentrations of IgM in the serum in terms of fixed effects of the diet treatments during the whole trial. There were tendencies (0.05 < *p* ≤ 0.10) for the cows supplemented with OTMs to have a greater concentration of IgA in their serum on d 30 and d 90 (Figure 4A). Cows supplemented with OTMs had higher concentrations of IgG in their serum than cows supplemented with ITMs (*p* ≤ 0.05) on d 30 (Figure 4B). Cows supplemented with OTMs had a higher concentration of IgM in their serum than cows supplemented with ITMs (*p* ≤ 0.05) on d 60 (Figure 4C). Meanwhile, the MHAC and AAC groups had higher concentrations of IgM in their serum on d 60 and d 90 (*p* ≤ 0.05) compared to those in the S group. Cows supplemented with OTMs had a higher concentration of ceruloplasmin than the cows supplemented with ITMs (*p* ≤ 0.05) on d 30 (Figure 4D).

### 3.4. Hoof Health

**Figure 5:**
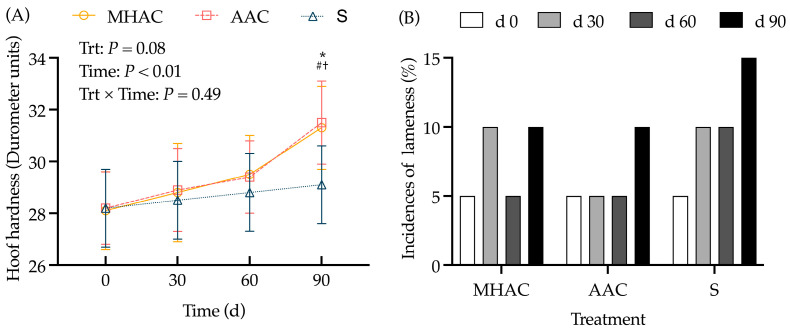
Effects of trace mineral sources on hoof hardness (**A**) and incidence of lameness (**B**) in lactating cows. Incidence of lameness (%) was calculated according to the number of lame cows divided by the total numbers in each group (*n* = 20). Cows were considered lame when their score was ≥3. Probability values for independent variables of interest: Trt = fixed effect of diet treatments; Time = fixed effect of sampling time; Trt × Time = interaction effect of diet treatments and sampling time. Within one day or one sampling, significant differences (*p* ≤ 0.05) were represented as follows: * ITM vs. OTM groups (contrast differences between inorganic trace mineral treatment (S, *n* = 20) and organic trace mineral treatments (MHAC and AAC, *n* = 40. # = MHAC vs. S; † = AAC vs. S.

Hoof health was assessed through histological examination using the hoof hardness test and the incidence of lameness. Through the whole trial, the MHAC and AAC groups had a tendency to show increased hoof hardness compared to that of the S group (*p* = 0.08). It is noteworthy that the cows supplemented with MHAC and AAC forms had a higher hoof hardness than the supplemented cows from the S group on d 90 (*p* < 0.05) (Figure 5A). Cows supplemented with the MHAC and AAC forms had a lower incidence of lameness, with a value of 10% (2/20), compared to that of the S group, with a value of 15% (3/20), on d 90 (Figure 5B).

## 4. Discussion

TMs are essential for immune function, hoof health, and reproductive performance in dairy cows [1,2,37]. Supplementation with TMs that have greater bioavailability, such as OTMs, has been shown to improve their health and production outcomes [5,13]. Here, we evaluated the effects of half-replacement of the supplementary sulfate sources of Cu, Mn, and Zn with the MHAC or AAC forms on the concentration of TMs in their body fluids, their blood immune biomarkers, and hoof health.

In the present study, the barns were not equipped with roughage intake control systems (RFID) to monitor the daily DMI individually, and the DMI was shown as the mean of the group DMI ± SD weekly. The replacement of the supplementary ITM forms of Cu, Mn, and Zn with OTM forms in the diet had various effects on DMI. Most previous studies have found no effect of trace mineral sources on DMI [14,38,39]. However, Mion et al. (2022) found a beneficial effect on DMI during the transition to lactation after supplementing cows with OTM sources, while the milk yield was not improved by the supplementation [15]. The cows supplemented with OTMs had a greater average milk yield on d 10, 15, and 90, a higher milk fat content on d 90, and a lower SCC on d 90 in the present study. Similar results have been observed in previous studies [20,21,23]. For instance, Cope et al. (2009) found that supplementation of an organically chelated form of Zn at the recommended level according to NRC (2001) [27] increased milk yield [39]. In contrast, Yasui et al. (2019) showed no difference in the milk yield, milk composition, or DMI when the STMs were completely replaced with OTMs (Cu, Mn, and Zn) in the diets of mid-lactation, multiparous cows fed for 6 weeks [40]. Similarly, Daniel et al. (2020) reported no differences in the DMI or milk, fat, and protein yields when replacing 30% of supplemental STMs (Cu, Mn, and Zn) with hydroxy-chloride TMs in the diets of mid-lactation cows [22]. The effect of the TM sources on milk yield has shown inconsistent results, likely due to variations in the TM sources, experimental designs, and data analysis methods. For example, statistically significant differences in the data were observed at some time points in the present study, while the interactions were not significant. This may have been to the inhomogeneous cows enrolled or a lack of statistical power.

Previous studies have found variable results in terms of dairy cow production response, especially in milk composition, to OTM sources [4,5,12,13]. Andrieu et al. (2008) found a decrease in the SCC in cows supplemented with TMs in the form of proteinates compared to ITMs [41]. Nocek et al. (2006) reported that replacing STMs (Co Cu, Mn, and Zn) with OTMs for two lactation cycles resulted in higher milk, fat, and protein production and a lower SCC [42]. Mion et al. (2022) evaluated the effects of the complete replacement of supplementary ITMs with OTMs in both pre- and postpartum diets on the feeding behavior, ruminal fermentation, rumination activity, energy metabolism, and lactation performance in dairy cows [15]. Their results showed that there was no effect of OTMs (complexed chelated Cu, Mn, and Zn) on the SCC. A meta-analysis of 22 studies on OTM supplementation found no significant changes in milk protein and fat percentages [43]. However, Mion et al. (2022) found that the percentage of protein in the milk was slightly greater under OTM supplementation [15]. The reasons for this may have been related to the supplemental level of total replacement with the TMs contributing to the effects of the TMs on tissue protein synthesis or microbial protein synthesis [12,44,45,46,47,48].

Some previous studies have suggested that their apparent digestibility and serum levels may not fully reflect the bioavailability of TMs, as the body regulates mineral absorption and excretion to prevent toxicity [49,50,51,52,53]. Further, liver biopsy has been proven to be a more reliable indicator of Cu status compared to plasma Cu concentrations [54]. These might also contribute to the marginally significant differences in the mineral concentrations in the serum in our study. It is, however, known according to the factorial prediction of the net requirements of Cu, Mn, and Zn recommended by NASEM (2021) that the changes in the net requirements would be much smaller than the changes in mineral intake [1]. For instance, the net requirement for Zn in a 700 kg cow is predicted [24] to increase from 79 to 300 mg/d between late gestation and a peak of milk production of 45 kg/d according to NASEM (2021) [1,34]. This additional requirement of 221 mg/d represents only 9% of the additional Zn intake in this study. This disproportionate change in supply as compared to the net requirements is even greater for Mn and Cu, as exports through the milk of these trace elements are low. The differences in the net requirements are predicted to be rather small, 11.8 to 12.0 mg/d for Cu and 2.1 to 3.2 mg/d for Mn, between the dry period and the peak of milk production. Such an increase in the net requirements represents less than 0.1% of additional intake of these minerals reported [34]. The lack of significant differences in the concentrations of Cu, Mn, and Zn in the feces and urine observed in our study might represent a lack of a difference in the true bioavailability for absorption among the three sources tested or the inability of our laboratory analyses to capture the differences in the concentration or bioavailability of these TMs post-absorption. Some studies have reported greater concentrations of Cu, Mn, and Zn in different tissues when supplementing with organic versus inorganic sources [43,55]. The long-term administration of OTMs led to increased levels of Cu, Mn, and Zn in the hoof keratin, which further demonstrated that OTMs have better bioavailability and better functions in hoof health [7]. Other studies have not found significant differences [20,56]. Complete replacement of supplementary ITMs with OTMs in both pre-and postpartum diets did not change the concentrations of Cu, Mn, and Zn; the activity of antioxidant biomarkers in the blood; or the overall incidence of postpartum clinical diseases but altered the concentrations of Co and Se in multiple specimens and reduced the incidence of lameness and postpartum metabolic problems [15]. It is noteworthy that the cows in this experiment were never fed a diet deficient in TMs, which may have made observing differences among treatments more difficult [27].

OTM supplementation improved hoof health by increasing the keratin-bound Cu, Mn, and Zn levels, likely due to their role in keratin formation and cartilage synthesis. Cu is essential for cross-linking keratin filaments, Mn supports proteoglycan synthesis, and Zn is crucial for cartilage remodeling [2]. Deficiencies in these minerals have been associated with hoof diseases, while OTM supplementation has been shown to enhance neutrophil function and reduce postpartum lameness [57].

Immune biomarkers also demonstrated positive responses to the OTMs. Cu, Mn, and Zn play important roles in the immune system [5,37]. The supplementation of OTMs in dairy diets affords higher bioavailability and is associated with beneficial effects on immune function [13,19,58]. Furthermore, immune function plays a critical role in the lameness of dairy cows by disrupting the production of hoof health [13,59]. We observed that the serum levels of ceruloplasmin, an acute-phase protein with antioxidant properties, were higher in the OTM-fed cows. Ceruloplasmin plays a role in resolving infections and restoring homeostasis during acute-phase responses [60,61]. Although ceruloplasmin levels are not always reliable indicators of Cu status because research [62] has shown that an increase in ceruloplasmin bound to circulating copper is not associated with changes in ceruloplasmin activity, our findings suggest that long-term OTM supplementation enhances both immune function and antioxidant capacity.

## 5. Conclusions

The half-replacement strategy showed that the methionine-hydroxy-analog-chelated Cu, Mn, and Zn and the amino-acid-complexed Cu, Mn, and Zn additives had similar effects. Half-replacement of the supplementary sulfate sources of Cu, Mn, and Zn with MHAC or AAC forms in the diet would improve the concentration of TMs in cows’ body fluids and their blood immune biomarkers, ultimately enhancing immune status and benefiting hoof health. Future research in this area may elucidate the optimal strategies for incorporating trace minerals into dairy cows’ diets further to maximize their performance and well-being and inform mineral supplementation strategies for other lactating animals.

## Figures and Tables

**Table 1:** Ingredient composition and nutrient levels (% of DM) of the final TMRs.

Item	Content
Corn silage	34.63
Alfalfa hay	10.49
Corn flour	22.60
Steam-flaked corn	1.11
Soybean meal	4.31
Cottonseed meal	8.48
Pelleted dried beet pulp	6.43
Sprayed corn bran	4.49
Molasses	3.38
Fatty acid calcium ^1^	0.70
Yeast culture	0.23
NaHCO_3_	0.95
Limestone	0.86
Ca (HCO_3_)_2_	0.34
MgO	0.20
NaCl	0.14
Urea	0.20
Montmorillonite	0.05
Mycotoxin adsorbent ^2^	0.07
Premix ^3^	0.34
Nutrient levels ^4^	
Crude protein	17.83
Starch	27.88
Ether extract	3.65
Neutral detergent fiber	29.87
Acid detergent fiber	17.10
Ash	7.73
Net energy for lactation (MJ/kg)	1.72

^1^ Megalac: Main component is palm fatty acid. ^2^ Biomin: Main ingredients are diatomaceous earth and yeast cell walls. ^3^ Three unique premixes were used (Table S1), differing in the source of the trace mineral supplements. The targeted levels of the premixes (per kg of DM): 2925.58 mg Cu, 7009.39 mg Mn, and 15,472.58 mg Zn, 248 mg I, 80 mg Se, 120 mg Co, 149 IU/kg Vitamin A, 50 IU/kg Vitamin D, and 24,752 IU/kg Vitamin E. ^4^ Nutrient levels were measured.

**Table 2:** Analyzed mineral content (mean ± SD, on a DM basis) of the final TMRs.

Item	Treatment ^1^		
	MHAC	AAC	S
Ca (g/kg)	10.15 ± 0.41	10.55 ± 0.55	10.05 ± 0.51
P (g/kg)	3.70 ± 0.18	4.05 ± 0.14	3.40 ± 0.20
Mg (g/kg)	4.90 ± 0.22	5.05 ± 0.21	4.85 ± 0.23
Na (g/kg)	8.20 ± 0.34	8.30 ± 0.41	8.35 ± 0.38
K (g/kg)	17.80 ± 1.18	17.55 ± 1.53	17.30 ± 1.72
Fe (mg/kg)	0.70 ± 0.05	0.68 ± 0.07	0.68 ± 0.07
Cu (mg/kg)	18.35 ± 1.12	20.45 ± 1.45	17.69 ± 1.63
Mn (mg/kg)	47.57 ± 2.71	44.53 ± 2.84	44.66 ± 3.26
Zn (mg/kg)	76.60 ± 5.58	77.60 ± 4.94	73.64 ± 5.79
Se (mg/kg)	0.50 ± 0.06	0.52 ± 0.04	0.51 ± 0.06

^1^ MHAC = replacing 50% of the sulfate form with 50% organic salts of trace minerals in methionine hydroxyl analog chelate form; AAC = replacing 50% of the sulfate form with 50% organic salts of trace minerals in amino acid complex form; S = 100% inorganic salts of trace minerals in sulfate form.

**Table 3:** Effects of trace mineral sources on the concentration of trace minerals in the serum, milk, hoof keratin, urine, and feces of lactating cows.

Item	Treatment ^1^			SEM	*p*-Value ^2^		
	MHAC	AAC	S		Trt	Time	Trt × Time
Serum							
Cu (mg/L)	0.81	0.82	0.79	0.014	0.07	0.71	0.90
Mn (μg/L)	2.76 ^a^	2.81 ^a^	2.71 ^b^	0.134	<0.01	<0.01	0.11
Zn (mg/L)	1.15	1.14	1.13	0.012	0.16	0.02	0.90
Milk (mg/kg)							
Cu	0.78 ^a^	0.76 ^a^	0.73 ^b^	0.015	0.04	0.53	0.26
Mn	0.09	0.09	0.08	0.002	0.36	0.02	0.29
Zn	4.24	4.14	4.12	0.156	0.94	0.73	0.96
Hoof keratin (mg/kg)							
Cu	8.75	8.63	7.98	0.072	0.08	0.59	0.43
Mn	5.78	5.72	5.48	0.043	0.09	0.42	0.37
Zn	57.61	57.49	55.73	0.175	0.18	0.46	0.55
Urine (mg/L)							
Cu	0.45	0.44	0.41	0.024	0.09	0.44	0.28
Mn	0.07	0.06	0.06	0.003	0.57	0.52	0.44
Zn	2.54	2.48	2.51	0.412	0.88	0.62	0.81
Feces (mg/kg)							
Cu	47.31	51.18	50.93	0.881	0.22	0.95	0.15
Mn	212.74	200.10	217.30	3.063	0.17	0.18	0.13
Zn	192.75	190.20	198.04	2.550	0.50	0.29	0.59

^1^ MHAC = replacing 50% of the sulfate form with 50% organic salts of trace minerals in methionine hydroxyl analog chelate form; AAC = replacing 50% of the sulfate form with 50% organic salts of trace minerals in amino acid complex form; S = 100% inorganic salts of trace minerals in sulfate form. ^2^ *p*-value for independent variables of interest: Trt = fixed effect of diet treatments; Time = fixed effect of sampling time; Trt × Time = interaction effect of diet treatments and sampling time. ^a,b^ The different lowercase letters of superscript indicate significant difference (*p* < 0.05).

## Data Availability

The data are contained within the article or Supplementary Materials.

## Supplementary Materials

The following supporting information can be downloaded at: https://www.mdpi.com/article/10.3390/ani15020271/s1, Figure S1: The concentrations of (A) Cu, (B) Mn, (C) Zn, (D) Ca, (E) P, (F) Mg, (G) Na, (H) K, and (I) Fe in the feed ingredients and basal TMRs without any kind of supplemental TMs; Table S1. Descriptive statistics of the experimental cows; Table S2: Composition of trace mineral and vitamin premix and the nutrient levels (on a DM basis); Table S3: Effects of trace mineral sources on milk composition and milk yield of lactating cows (% unless otherwise noted).
