# Supplementary material for: Effects of Replacing Inorganic Sources of Copper, Manganese, and Zinc with Different Organic Forms on Mineral Status, Immune Biomarkers, and Lameness of Lactating Cows
Source: Animals (Basel). 2025 Jan 19;15(2):271. doi: 10.3390/ani15020271 (PMC11763283; doi:10.3390/ani15020271)
Supplement: Supplementary file 1 [file animals-15-00271-s001.zip › animals-3376466-supplementary.pdf]

animals

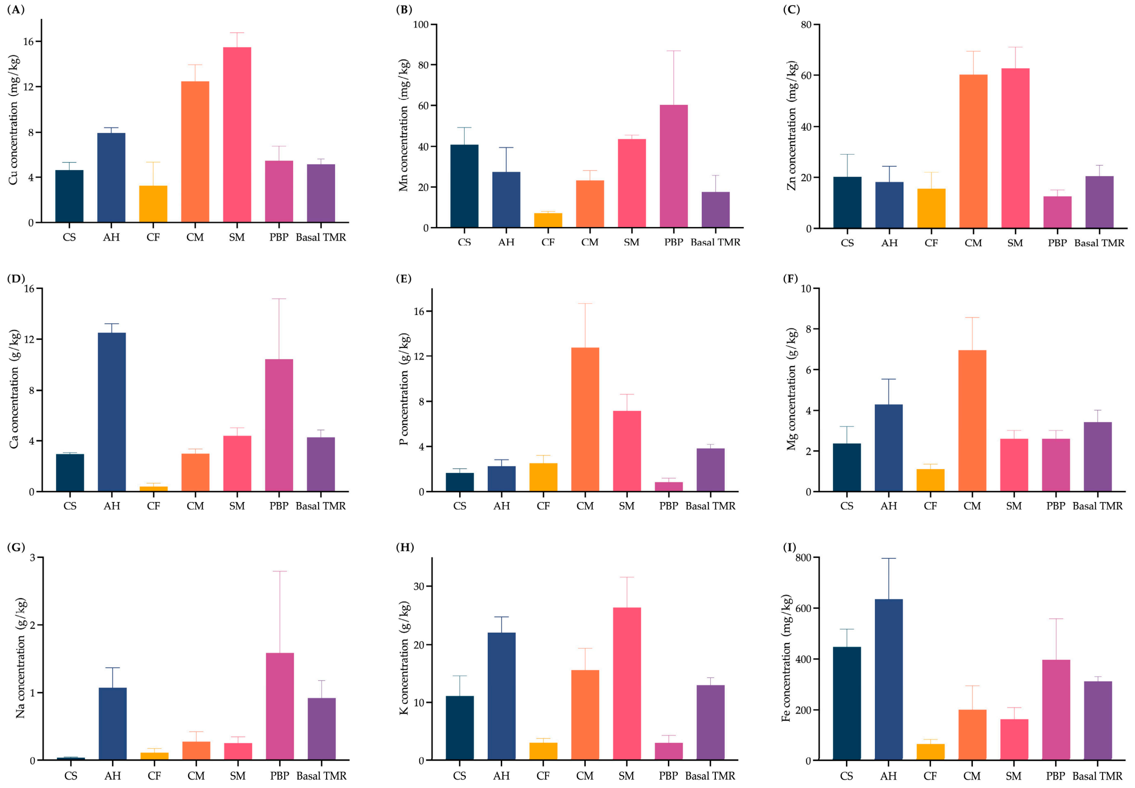

Figure S1. The concentrations of (A) Cu, (B) Mn, (C) Zn, (D) Ca, (E) P, (F) Mg, (G) Na, (H) K, and (I) Fe in the feed ingredients and basal TMR without any kind of supplemental TM. CS = corn silage; AH = alfalfa hay; CF = corn flour; CM = soybean meal; PBP = pelleting dried beet pulp; Basal TMR = basal TMR without any kind of supplemental TM. Data are expressed as means ± standard deviation. Each feed ingredient and the basal TMR samples were collected 3 times, and each analysis was conducted in 2 replicates.

Table S1. Descriptive statistics of the experimental cows

Groups	Items	n	Minimum	Maximum	Mean	standard deviation
Cows enrolled	Days in milk	60	101.0	250.0	157.7	26.1
	Body weight	60	520.8	752.6	664.5	52.0
	Milk yield	60	21.5	45.4	31.7	6.5
S group	Days in milk	20	101.0	208.0	152.7	22.7
	Body weight	20	520.8	721.2	653.0	54.0
	Milk yield	20	21.9	45.4	32.1	6.8
MHAC group	Days in milk	20	105.0	250.0	162.5	27.8
	Body weight	20	532.5	748.5	668.7	49.2
	Milk yield	20	21.7	43.7	31.8	6.5
AAC group	Days in milk	20	103.0	235.0	157.9	27.9
	Body weight	20	565.1	752.6	672.0	53.4
	Milk yield	20	21.5	43.6	31.3	6.4

Table S2. Composition of trace mineral and vitamin premix and the nutrient levels (DM basis)

Item	MHAC	AAC	S
Inorganic Cu (mg/kg)[1]	0.59	0.59	1.17
Organic Cu (mg/kg)[2]	1.22	1.46	0.00
Inorganic Mn (mg/kg)[3]	1.10	1.10	2.20
Organic Mn (mg/kg)[4]	2.92	4.38	0.00
Inorganic Zn (mg/kg)[5]	2.24	2.24	4.48
Organic Zn (mg/kg)[6]	6.45	6.45	0.00
$Ca(IO_3)_2$ (1% I) (mg/kg)	2.48	2.48	2.48
Na_2SeO_3 (1% Se) (mg/kg)	0.48	0.48	0.48
$C_3H_7NO_2Se$ (1% Se) (mg/kg)	1.60	1.60	1.60
$CoSO_4$ (1% Co) (mg/kg)	1.20	1.20	1.20
Vitamin A (IU/kg)	0.30	0.30	0.30
Vitamin D (IU/kg)	0.10	0.10	0.10
Vitamin E (IU/kg)	4.95	4.95	4.95
Rice chaff (%)	4.73	3.03	11.39
Limestone flour (%)	69.64	69.64	69.64
Total（%)	100	100	100
Nutrient levels (otherwise noted)			
Total Cu (mg/kg)	2925.58	2925.58	2925.58
Inorganic Cu (mg/kg)	1462.79	1462.79	2925.58
Organic Cu (mg/kg)	1462.79	1462.79	0.00
Total Mn (mg/kg)	7009.39	7009.39	7009.39
Inorganic Mn (mg/kg)	3504.70	3504.70	7009.39
Organic Mn (mg/kg)	3504.70	3504.70	0.00
Total Zn (mg/kg)	15472.58	15472.58	15472.58
Inorganic Zn (mg/kg)	7736.29	7736.29	15472.58
Organic Zn (mg/kg)	7736.29	7736.29	0.00
I (mg/kg)	248.00	248.00	248.00
Se (mg/kg)	80.00	80.00	80.00
Co (mg/kg)	120.00	120.00	120.00
Vitamin A (IU/kg)	149.00	149.00	149.00
Vitamin D (IU/kg)	50.00	50.00	50.00
Vitamin E (IU/kg)	24752.00	24752.00	24752.00

[1] MHAC = replacing 50% sulfate form with 50% organic salts of trace minerals in methionine hydroxyl analog chelate form; AAC = replacing 50% sulfate form with 50% organic salts of trace minerals in amino acid complex form; S = 100% inorganic salts of trace minerals in sulfate form.

[2] Inorganic copper in MHAC, AAC and S are all sulfate copper ($CuSO_4$-5H2O, copper content 25.0%); [3] The organic copper in MHAC and AAC are MHAC-Cu (copper content 12%) and AAC-Cu (copper content 10.0%), respectively.

[4] Inorganic manganese in MHAC, AAC and S are all sulfate manganese ($MnSO_4$-4H2O, manganese content 31.8%).

[5] The organic manganese in MHAC and AAC are MHAC-Mn (manganese content 12.0%) and AAC-Mn (manganese content 8.0%), respectively.

[6] Inorganic zinc in MHAC, AAC and S are all sulfate zinc ($ZnSO_4$-7H2O, zinc content 34.5%); [6] Organic zinc in MHAC and AAC are MHAC-Zn (zinc content 12.0%) and AAC-Zn (zinc content 12.0%), respectively.

Table S3. Effects of trace mineral sources on milk composition and milk yield of lactating cows (% unless otherwise noted)

Items (%)	Treatments [1]			SEM	*P*-value [2]		
	MHAC	AAC	S		Trt	Time	Trt×Time
Milk fat	4.02	4.00	3.98	0.153	0.97	<0.01	0.99
Milk protein	3.64	3.64	3.63	0.034	0.98	0.73	0.98
Milk lactose	4.84	4.78	4.83	0.098	0.55	<0.01	0.46
Milk solid	13.69	13.44	13.4	0.172	0.41	0.03	0.7
Milk urea nitrogen (mg/dL)	13.97	14.08	14.16	0.243	0.85	<0.01	0.97
Milk fat yield (kg/d)	1.21	1.19	1.20	0.038	0.96	0.06	0.99
Milk protein yield (kg/d)	1.06	1.03	1.04	0.026	0.92	<0.01	0.98
4% fat corrected milk (kg/d)[3]	29.66	29.46	29.88	3.765	0.96	<0.01	0.29

[1] MHAC = replacing 50% sulfate form with 50% organic salts of trace minerals in methionine hydroxyl analog chelate form; AAC = replacing 50% sulfate form with 50% organic salts of trace minerals in amino acid complex form; S = 100% inorganic salts of trace minerals in sulfate form. [2] *P*-value for independent variables of interest: Trt = fixed effect of diet treatments; Time = fixed effect of sampling time; Trt × Time = interaction effect of diet treatments and sampling time. [3] 4% fat corrected milk was calculated as milk yield (kg/d) × [0.377 + 0.116 × fat (%) + 0.06 × protein (%)]
